# Longitudinal, cross-cohort comparison of physical activity patterns in Chinese mothers and children

**DOI:** 10.1186/1479-5868-9-39

**Published:** 2012-04-03

**Authors:** Tracy Dearth-Wesley, Penny Gordon-Larsen, Linda S Adair, Bing Zhang, Barry M Popkin

**Affiliations:** 1 Department of Nutrition and Carolina Population Center, University of North Carolina at Chapel Hill, Chapel Hill, NC, USA; 2 National Institute of Nutrition and Food Safety, Chinese Center for Disease Control and Prevention, 29 Nanwei Rd, Beijing, 100050, China

**Keywords:** Physical activity, Sedentary behavior, China, Longitudinal, Mothers, Children, Socioeconomic

## Abstract

**Background:**

There is limited evidence comparing adult and child physical activity (PA) trends and examining parent–child PA associations within a newly industrialized country setting. PA research within a newly industrialized country setting is particularly important given the negative effects of rapid urbanization, socioeconomic growth, and technological advances on PA behaviors. The purpose of our study was to examine trends and associations in PA behaviors in Chinese mother-child pairs and to investigate relationships between PA behaviors and socioeconomic variables in this dyad.

**Methods:**

We studied PA behaviors in 2 separate cohorts of mother-child pairs (n = 353) followed over a 2–4 year time period using longitudinal data from the China Health and Nutrition Survey (2000 Cohort: 2000–2004; 2004 Cohort: 2004–2006). Comparable mother-child PA behaviors included total metabolic equivalent hours per week (MET-hrs/wk) from active commuting, leisure-time sports, and sedentary behaviors. Logistic regression models were used to examine associations between mother and child PA and relationships between PA behaviors and socioeconomic variables.

**Results:**

Children experienced increases in active commuting and leisure-time sports activities with increasing child age, whereas mothers experienced temporal declines in active commuting and minimal change in leisure-time sports activity. Sedentary behavior was high for children and mothers over time. Mother-child associations were positive for active commuting and leisure-time sports activities and negative for sedentary behavior (*P* < 0.05). Maternal education was associated with a greater likelihood of high leisure-time sports activity and high sedentary behavior in mothers but not in children (*P* < 0.05).

**Conclusion:**

Efforts to reduce sedentary behavior in Chinese mothers and children are imperative. While increased leisure-time and active commuting activities in children is encouraging, continued PA promotion in children and more intensive efforts to promote leisure-time sports and active commuting in mothers is needed.

## Background

Continued global reductions in occupational and domestic activities and increases in passive commuting are inevitable based on rapid urbanization, socioeconomic growth, and technological advances [1-4], thus leisure-time physical activity (PA) and active commuting are increasingly important for chronic disease prevention. However, one in five adults worldwide does not meet the global recommendations for PA and is considered physically inactive [5]. Less is known about global PA patterns in children, particularly in newly industrialized and developing countries experiencing rapid socioeconomic growth [6].

More research on PA patterns in children is needed, and integration of this research with adult PA patterns is important given the positive relationships between parent and child sports participation, vigorous activity and inactivity [7-12]. Children with two active parents are more likely to participate in sport as compared to children with inactive parents; parental inactivity strongly predicts child inactivity [7-10]. While these studies were conducted in developed countries and were mostly cross-sectional, some longitudinal research has shown that the parent–child PA relationship weakens or no longer exists with increasing child age [13-15]. Additional longitudinal research is needed to more thoroughly evaluate the relationship between parent and child PA over time within a newly industrialized country setting.

Past research on parent–child PA dynamics in China found parental encouragement and role modeling to be positively related to child’s attraction to and participation in PA [16-18]. Parental influence on child PA in China may be explained in part by parental “training” (guan), which is a more controlling parenting style stressing hard work and self-discipline [16]. While systematic comparison of PA patterns in Chinese mothers versus children has not been done, past research reported a 32% decline in the average weekly PA for Chinese adults from 1991 to 2006 [3] and rapid increases in sedentary behavior, namely screen time, among Chinese youth over the last decade [19]. While trends toward decreased PA and increased sedentary behavior are accelerated in newly industrialized and developing countries experiencing rapid socioeconomic growth, urbanization and technological advances [1-4], decreased PA among adults and increased sedentary behavior among youth are also well-documented in developed countries [20-22].

Using longitudinal data from the China Health and Nutrition Survey (CHNS), we examined PA behaviors in 2 separate cohorts of mother-child pairs followed over a 2–4 year time period. Comparable mother-child PA behaviors included active commuting, leisure-time sports activity, and sedentary behavior. Use of the two cohorts permitted examination into how mother and child PA changes across different time periods experiencing rapid socioeconomic development and urbanization [23]. Our primary study objectives were (1) to compare PA trends in mothers and children, (2) to examine associations between mother and child PA behaviors over time and (3) to examine the relationships between PA behaviors and socioeconomic variables in mothers and children.

## Methods

### Data and subjects

We used longitudinal data from the CHNS. The CHNS began in 1989 with subsequent surveys every 2–4 years. A multistage, randomized cluster design was utilized to survey around 4,400 households and roughly 19,000 individuals from 9 Chinese provinces that vary in geography, socioeconomic growth, and health indicators. No adjustments for sampling design were done, as past research found that adjustments were necessary only when community level factors were examined [24]. Additional CHNS details are available in previous publications [25,26]. The study met the standards for the ethical treatment of participants and was approved by the Institutional Review Boards of the University of North Carolina at Chapel Hill and the Institute of Nutrition and Food Safety, Chinese Center for Disease Control and Prevention.

Our study sample included 2 separate cohorts of biological mother-child pairs followed over a 2 or 4-year time period (2000 Cohort: 2 measurement occasions 2000 & 2004; 2004 Cohort: 2 measurement occasions 2004 & 2006). Children in the 2000 Cohort were 6–8 years of age at baseline; children in the 2004 Cohort were 7–9 years of age at baseline. The cohorts were determined based on 2 factors: (a) The cohorts are an extension of previous dietary research in the same mother-child pairs [27]; (b) The sample was restricted to children who remained <12 years of age throughout the study, since children ≥12 years of age leave primary school and enter middle school (i.e., potential for different commuting distances).

The analytic sample included mother-child pairs who had complete PA data at both measurement occasions. Complete PA data was defined as mother and child measurements for active commuting and leisure-time sports at both measurement occasions and measurements for sedentary behavior at the second measurement occasion. Since data on sedentary behavior in adults was first collected in the 2004 CHNS, only cross-sectional mother-child comparison of sedentary behavior was feasible. A total of 872 mother-child pairs had at least some mother or child PA data at baseline, while 353 mother-child pairs (40%) had complete PA data. Mother-child pairs with incomplete PA data did not significantly differ from the analytic sample with respect to baseline sociodemographic and PA variables.

### Measures

CHNS data were collected using structured questionnaires administered by trained field staff to all household members. Parents or primary caregivers completed or assisted with the completion of surveys for children <10 years. Details concerning the socioeconomic measures (i.e., residence, income, maternal education) have been previously published [25,26].

Children and mothers were asked about their participation and weekly time spent in commuting to and from school or work and in specific groups of sports activities and sedentary behaviors. Active commuting included biking or walking to school or work. Leisure-time sports were grouped into 4 main categories: gymnastics, track and field/swimming, ball sports (e.g., tennis, basketball, soccer), and other sports (e.g., martial arts, tai chi). Sedentary behavior consisted of 4 main categories: TV/DVD watching, board/video games, extracurricular reading and writing, and computer usage. While children were asked about their participation and time spent doing homework, the measure was not included in the sedentary category to enable mother-child comparability of sedentary behavior.

The main PA variables were total metabolic equivalent hours per week (MET-hrs/wk) from active commuting, leisure-time sports, and sedentary behaviors. First, each reported activity was assigned a MET value using the Compendium of Energy Expenditures for Youth for children and the Compendium of PA for mothers [28,29]. Average MET values were used for activity categories such as ball sports or other sports. The MET value for each activity was then multiplied by the total time spent per week (hrs/wk) in the activity, resulting in the MET-hrs/wk measurement. Implausible high values were examined within the context of each activity domain and its categories and replaced with plausible maximum values using domain and category-specific criteria. For example, leisure-time activities exceeding 60 hours/week were replaced with the plausible maximum value of 60 hours/week. Plausible maximum values were created using previous PA research in China and other supporting documents [3,30-33],

High versus low activity categories based on the World Health Organization’s Global PA Recommendations or on time-based cut-points were created to facilitate interpretation of the findings and applicability to PA research, policy, and intervention efforts [34]. High active commuting was defined as the MET-hrs/wk equivalent of ≥30 minutes/day of biking or walking [3,35]. High leisure-time sports activity for children was defined as the MET-hrs/wk equivalent of at least 60 minutes of moderate- to vigorous-intensity PA daily, with vigorous-intensity PA at least 3 times per week [34]. High leisure-time sports activity for mothers was defined as the MET-hrs/wk equivalent of at least 150 minutes of moderate-intensity PA per week, at least 75 minutes of vigorous-intensity PA per week, or an equivalent combination of moderate- to vigorous-intensity PA [34]. While we acknowledge that the WHO Global PA Recommendations include activity from leisure-time, commuting, occupational, and domestic domains, these recommendations provided a feasible cut-point for our high vs. low leisure-time sports category. High sedentary behavior for mothers and children was defined as the MET-hrs/wk equivalent of ≥2 hours/day of sedentary behavior [36].

### Data analysis

All analyses were conducted using Stata version 11.0 (Stata Corporation, College Station, TX, USA). Significant differences between the cohorts with respect to baseline characteristics were examined using chi-squared and t tests. Mother-child comparison of changes in PA over time was done using the Wilcoxon signed rank test and the average annual change measure. The Wilcoxon signed rank test was used to examine differences in the total MET-hrs/wk from each activity domain or activity category from baseline to follow-up. Average annual changes in PA were calculated by subtracting the average MET-hrs/wk from the first time point from the average MET-hrs/wk from the second time point and then dividing the result by the difference in the years between the 2 survey time points. Average annual changes were determined separately in each cohort in order to examine how PA changes in mothers and children with increasing child age.

To examine associations between mothers’ and children’s PA or sedentary behavior, we used logistic regression models. Separate logistic regression models were run at baseline and follow-up using pooled cohort data. These models examined how high (versus low) maternal activity was associated high child activity for active commuting, leisure-time sports, and sedentary categories. Logistic regression was done given the large percentage of mothers reporting no active commuting or leisure-time sports activities (36% and 93%, respectively). Child’s gender was identified for potential effect measure modification (EMM) (i.e., exposure-outcome relationship varies within levels of a third variable), as previous research has shown that mother-child PA associations vary by child’s gender [7,8]. However, comparison of stratum-specific estimates and results from Breslow-Day tests of homogeneity did not indicate that child’s gender modified the mother-child PA or sedentary behavior association. Child’s gender was next examined as a confounder. Child’s gender, socioeconomic and cohort variables, and maternal and child age were included as confounders based on supporting research and our examination of the relations between covariates and mother and child PA/sedentary behavior [4,13-15,21,30,32,37,38].

To examine cross-sectional associations between socioeconomic variables with PA or sedentary behaviors in mothers and children, we used logistic regression models. Logistic regression models examined how socioeconomic variables were associated with high active commuting, leisure-time sports, or sedentary behaviors. Models for active commuting and leisure-time sports activities used data from both measurement occasions, and models for sedentary behavior used data at follow-up. Separate models were run in mothers and children for active commuting, leisure-time sports, and sedentary categories. Child’s gender was again assessed for EMM in all child models but was not found to modify the associations based on stratum-specific estimates comparisons and Breslow-Day tests of homogeneity. Child models controlled for cohort, child’s gender and child’s age and maternal models controlled for cohort and maternal age based on supporting research and relations among covariates, socioeconomic variables, and PA/sedentary behavior[3,30,39,40].

## Results

Significant differences between the cohorts were found for child’s age, mother’s age and annual household income (*P* < 0.001) (Table 1). Children and mothers in the 2000 Cohort were younger than children and mothers in the 2004 Cohort. The mean household income inflated to 2006 yuan currency values was higher in the 2004 Cohort versus the 2000 Cohort.

**Table 1:** **Baseline characteristics of mother-child pairs by cohort**^**1,2**^

	**2000 Cohort**	**2004 Cohort**
Survey years	2000, 2004	2004, 2006
*N*	167	186
Child’s gender, *% male*	55.7	51.1
Child’s age, y*	7.4 ± 0.9	8.4 ± 0.9
Mother’s age, y*	32.8 ± 3.5	34.6 ± 3.8
Mother’s education, *%*		
None/primary school	52.5	42.5
Middle school	34.4	41.3
High school	8.7	8.4
College, technical or higher	4.4	7.8
Annual household income, *yuan*^3*^	16045 ± 14267	20740 ± 18733
Household residence, *% rural*	76.7	76.3

China Health and Nutrition Survey.^**1,2**^

*Different between cohorts, *P* < 0.001.

^1^Values are mean ± SD or percentage.

^2^Data were missing for mother’s age (n = 11), mother’s education (n = 14), and household income (n = 10).

^3^Annual household income inflated to 2006 yuan currency values.

### Descriptive

Whereas active commuting MET-hrs/wk increased for children over time, we found temporal declines in MET-hrs/wk for mothers (*P* < 0.001) (Table 2). Children reported increases in MET-hrs/wk from biking and decreases in MET-hrs/wk from walking, while mothers reported decreases in MET-hrs/wk from both activities (*P* < 0.05). The proportion engaged in high active commuting increased for children from baseline to follow-up but decreased for mothers (Table 3).

**Table 2:** **MET-hrs/wk for total and categories of commuting, leisure-time sports, and sedentary behaviors and average annual changes in mothers and children by cohort**^**1**^

	**2000 Cohort (Baseline 2000; Follow-up 2004)**						**2004 Cohort (Baseline 2004; Follow-up 2006)**					
	**Children**			**Mothers**			**Children**			**Mothers**		
	**Baseline**	**Follow-up**	**Annual change**	**Baseline**	**Follow-up**	**Annual change**	**Baseline**	**Follow-up**	**Annual change**	**Baseline**	**Follow-up**	**Annual change**
Total commuting^2^	6.5 (9.8)*	8.6 (11.5)	0.5	7.2 (8.3)*	5.9 (12.3)	−0.3	8.2 (10.9)*	8.5 (8.6)	0.2	7.1 (15.2)*	5.0 (7.7)	−1.1
Walking	6.0 (8.6)*	5.6 (11.1)		4.5 (5.9)*	3.5 (5.7)		6.2 (10.7)*	5.9 (6.6)		3.9 (10.1)*	3.3 (5.2)	
Biking	0.7 (5.8)*	3.1 (6.4)		3.3 (5.6)*	3.2 (13.3)		2.1 (5.7)*	2.6 (7.7)		4.3 (13.6)*	2.1 (6.0)	
Total leisure-time sports^2^	7.5 (17.9)^†^	21.1 (48.2)	3.4	1.8 (9.6)*	2.2 (14.4)	0.1	10.6 (31.2)	14.7 (33.2)	2.1	2.2 (11.9)*	3.2 (11.1)	0.5
Gymnastics	0.5 (2.5)*	1.2 (9.4)		<0.1 (0.4)*	0.8 (8.3)		1.4 (6.7)*	1.7 (7.7)		0.4 (5.0)*	0.7 (6.5)	
Track, swimming	4.5 (14.7)*	8.3 (26.7)		0.3 (3.1)*	0.2 (2.3)		5.0 (23.6)*	4.4 (15.2)		1.0 (8.4)*	1.0 (5.7)	
Ball sports	1.5 (7.6)*	8.8 (22.5)		0.2 (1.7)*	0.9 (8.8)		3.1 (15.0)*	6.7 (20.9)		0.7 (7.0)*	0.8 (4.7)	
Other sports	1.1 (3.7)*	2.8 (10.2)		1.3 (8.1)*	0.1 (1.8)		1.1 (6.0)*	2.0 (7.2)		0.1 (1.4)*	0.5 (4.1)	
Sedentary^2^		21.4 (18.2)			15.5 (12.6)			22.6 (11.6)			15.3 (10.5)	
TV/DVD watching		12.7 (10.8)			13.3 (9.4)			14.4 (8.9)			12.8 (8.2)	
Board/video games		3.4 (8.2)			0.1 (1.4)			2.2 (4.2)			0.0 (0.0)	
Reading/writing		4.5 (5.2)			1.7 (4.8)			5.0 (5.2)			1.3 (3.7)	
Computer usage		0.8 (3.3)			0.4 (2.4)			1.0 (3.6)			1.3 (5.4)	
Homework^3^		13.3 (11.7)						3.5 (9.9)				

China Health and Nutrition Survey^**1**^

*Different between baseline and follow-up, *P* < 0.05.

^1^Values are means (SD).

^2^Categories of commuting, leisure-time sports, and sedentary behaviors do not sum to the total MET-hrs/wk, since a child or mother can report MET-hrs/wk from more than one category.

^3^Homework not included in total sedentary behavior.

**Table 3:** **Proportion with high or low activity levels for commuting, leisure-time sports, and sedentary behavior for mothers and children by cohort**^**1**^

	**2000 Cohort (Baseline 2000; Follow-up 2004)**				**2004 Cohort (Baseline 2004; Follow-up 2006)**			
	**Children**		**Mothers**		**Children**		**Mothers**	
	**Baseline**	**Follow-up**	**Baseline**	**Follow-up**	**Baseline**	**Follow-up**	**Baseline**	**Follow-up**
Commuting^2^								
Low activity	94.0	88.0	78.4	89.3	89.8	86.0	83.3	88.2
High activity	6.0	12.0	21.6	10.2	10.2	14.0	16.7	11.8
Leisure-time sports^3^								
Low activity	90.8	79.1	88.3	96.3	88.4	81.8	95.6	91.2
High activity	9.2	20.9	11.7	3.7	11.6	18.2	4.4	8.8
Sedentary^4^								
Low sedentary behavior		41.1		71.2		50.8		71.8
High sedentary behavior		58.9		28.8		49.2		28.2

China Health and Nutrition Survey.^**1**^

^1^Values are percentages.

^2^Low commuting activity = MET-hrs/wk equivalent of <30 minutes/day of biking/walking; high commuting activity = MET-hrs/wk equivalent of ≥30 minutes/day of biking/walking.

^3^Low leisure-time sports = mother or child does not meet the WHO Global PA Recommendation; high leisure-time sports activity = mother or child meets the WHO Global PA Recommendations.

^4^Low sedentary behavior = MET-hrs/wk equivalent of <2 hrs/day of sedentary behavior; high sedentary behavior = MET-hrs/wk equivalent of ≥2 hrs/day of sedentary behavior.

Temporal increases in MET-hrs/wk from leisure-time sports activity were found for mothers and children, but average annual changes for children were at least 4 times greater than those found for mothers (Table 2). With respect to categories of leisure-time sports activity, the most notable increases from baseline to follow-up for children were found for ball sports (e.g., MET-hrs/wk for ball sports increased from 1.5 to 8.8 for children in the 2000 Cohort and from 3.1 to 6.7 for children in the 2004 Cohort). The proportion reporting high leisure-time sports activity increased over time for children but was less consistent over time for mothers across the cohorts (Table 3).

Sedentary MET-hrs/wk for children were roughly 6–7 MET-hrs/wk higher as compared to that of mothers, which translates into 4–5 more hrs/wk of sedentary behavior for children versus mothers (Table 2). Sedentary behavior for mothers and children was primarily comprised of TV/DVD watching. A higher proportion of children versus mothers engaged in high sedentary behavior (Table 3).

### Modeling associations between mothers’ and children’s PA or sedentary behavior

High active commuting in mothers was associated with an increased likelihood of high active commuting in children at baseline and follow-up (*P* < 0.05) (Table 4). For leisure-time sports activity, children with mothers engaged in high sports activity had a greater likelihood of high sports activity at baseline (*P* < 0.05), but this relationship did not persist at follow-up. High maternal sedentary behavior was inversely associated with high sedentary behavior in children (*P* < 0.05).

**Table 4:** **Mother-child associations for high commuting, leisure-time sports or sedentary behaviors at baseline and follow-up**^**1,2**^

	**Outcome: high activity/behavior in children for corresponding activity/behavior category**	
**Maternal exposure**	**Baseline**	**Follow-up**
Commuting		
Low activity	1.00	1.00
High activity	2.72 (1.08, 6.87)^‡^	3.13 (1.37, 7.16)^†^
Leisure-time sports		
Low activity	1.00	1.00
High activity	3.61 (1.14, 11.41)^‡^	0.87 (0.26, 2.93)
Sedentary		
Low sedentary behavior	*	1.00
High sedentary behavior	*	0.58 (0.35, 0.97)^‡^

China Health and Nutrition Survey.^**1,2**^

*Sedentary behavior available at follow-up only for mothers.

^†^*P* < 0.01, ^‡^*P* < 0.05.

^1^Separate models for each outcome variable (child commuting, leisure-time sports, and sedentary).

^2^Odds Ratio (95% CI) controlled for child’s gender, child and maternal age, maternal education,residence, income, and cohort.

### Modeling associations between socioeconomic variables with PA or sedentary behavior in mothers and children

We found few significant associations between socioeconomic factors and high active commuting, leisure-time sports, or sedentary behaviors in mothers and children. Rural children were less likely than their urban counterparts to engage in high active commuting (*P* < 0.01) (Table 5). Mothers with a technical, college, or higher education had a greater likelihood of high leisure-time sports activity and high sedentary behavior versus mothers with none or a primary school education (*P* < 0.05).

**Table 5:** **Cross-sectional examination of socioeconomic correlates of high commuting, leisure-time sports, and sedentary behaviors in mothers and children**^**1,2,3**^

	**Outcome: high level commuting activity**		**Outcome: high level sports activity**		**Outcome: high level sedentary behavior**	
**Exposure**	**Children**	**Mothers**	**Children**	**Mothers**	**Children**	**Mothers**
Household residence						
Urban	1.00	1.00	1.00	1.00	1.00	1.00
Rural	0.43 (0.25, 0.75)^†^	0.74 (0.45, 1.24)	0.97 (0.57, 1.66)	0.67 (0.33, 1.35)	0.64 (0.37, 1.12)	0.63 (0.35, 1.13)
Household income						
Low	1.00	1.00	1.00	1.00	1.00	1.00
Middle	1.30 (0.66, 2.54)	0.90 (0.52, 1.56)	1.55 (0.83, 2.89)	0.70 (0.29, 1.69)	0.87 (0.48, 1.59)	0.86 (0.43, 1.45)
High	0.81 (0.39, 1.68)	0.83 (0.47, 1.47)	1.53 (0.82, 2.88)	0.58 (0.23, 1.46)	0.86 (0.47, 1.58)	1.20 (0.61, 1.46)
Maternal education						
None/primary	1.00	1.00	1.00	1.00	1.00	1.00
Middle school	1.52 (0.85, 2.71)	0.86 (0.53, 1.39)	1.57 (0.94, 2.60)	0.82 (0.35, 1.91)	0.71 (0.43, 1.19)	1.17 (0.66, 2.09)
High school	1.06 (0.39, 2.89)	0.68 (0.28, 1.64)	1.54 (0.69, 3.43)	3.32 (1.21, 9.19)^‡^	0.45 (0.19, 1.07)	1.24 (0.49, 3.16)
Technical, college or higher	1.37 (0.49, 3.85)	0.51 (0.17, 1.55)	1.40 (0.55, 3.61)	9.34 (3.56, 24.63)^‡^	0.56 (0.21, 1.50)	4.45 (1.61, 12.30)^†^

China Health and Nutrition Survey.^**1,2,3**^

^†^*P* < 0.01, ^‡^*P* < 0.05.

^1^Odds Ratio (95% CI).

^2^Separate models for mothers and children for each outcome variable (high commuting, leisure-time sports, and sedentary). Child models controlled for cohort, child’s age and gender. Maternal models controlled for cohort and maternal age.

^3^Models for commuting and leisure-time sports activities used data from both measurement occasions, and models for sedentary behavior used data at follow-up.

## Discussion

Using unique maternal and offspring PA and sedentary behavior data from a country undergoing major economic, social, and environmental change, we found disparate trends in mothers and their children. Whereas active commuting and leisure-time sports activities increased for children over time, active commuting declined and there was minimal change in leisure-time sports activity for mothers over time. In general, sedentary behavior was high across mothers and their children over time. Overall there were positive mother-child associations for active commuting and leisure-time sports activities and a negative mother-child association for sedentary behavior.

Temporal shifts toward increased leisure-time sports activity were more pronounced in children versus mothers. Increased leisure-time sports activity in children is hypothesized to result in part from national PA initiatives, such as the Nationwide Physical Fitness Program. The 15-year-long program began in 1995 and promoted PA in Chinese youth through the establishment of juvenile sports clubs and new public sporting facilities [41,42]. Concurrent to national initiatives is the growing popularity of competitive sports like basketball, which is also supported by the government construction of 600,000 basketball courts across the country [43,44]. While increased leisure-time sports activity in Chinese youth is encouraging, minimal sports activity in mothers coupled with the continued modernization of occupational and domestic activities necessitates more intensive PA promotion efforts for Chinese adults.

Our study documented a significant positive mother-child relationship for high leisure-time sports activity at baseline but no association at follow-up. This finding is consistent with previous research showing decreased associations between parent and child sports activity with increasing child age [13-15]. While decreased mother-child associations with increasing child age may be consequent of more PA promotion efforts targeted at children, it also suggests that parental PA patterns may be less influential on child PA patterns with increasing child age. Further longitudinal investigation into how other parental factors (e.g., parental beliefs and support) relate to child PA patterns is needed, particularly since cross-sectional research has shown these factors to be significantly correlated with child PA [12,45-47]. Improved understanding into how these factors influence child PA patterns is important for more effective family-based interventions promoting child PA.

The association between socioeconomic factors with PA and sedentary behaviors differed for mothers and children. Maternal education was significantly associated with a greater likelihood of high leisure-time sports activity and high sedentary behavior in mothers, similar to other research in China [3,32,37]. However, maternal education was not significantly associated with PA in children. This is similar to findings from a review of PA correlates in youth (4–12 years), which did not find parental education to be a significant correlate of PA [48]. Additional research examining well-established correlates of PA in youth (e.g., parental support, self efficacy, and physical environment factors) is needed in countries experiencing rapid socioeconomic and environmental changes [12,46,48].

A large proportion of mothers and children engaged in high levels of sedentary behavior. More than half of the children in our study reported ≥2 hrs/day of sedentary behavior in the 2000 and 2004 cohorts, mostly from TV/DVD watching. Our finding suggests a trend toward increased sedentary behavior among Chinese youth, since previous CHNS research found only 8% of Chinese youth (6–18 years) watched TV ≥2 hrs/day in 1997 [30]. Increased sedentary behavior in children and mothers is correlated with greater TV ownership in Chinese households [49]. Household TV ownership in China increased from 63% in 1989 to more than 95% in 2004 [21]. In 2006, 98% of Chinese households with children had a color TV [26]. High sedentary behavior, namely hours of TV watching, has been linked to detrimental health implications in children and adults (e.g., greater body mass index, increased cardiovascular disease risk) [50-52]. These negative health implications also pose a potentially large economic burden [53], thus targeted public health policy and interventions aimed at limiting sedentary behavior in the Chinese population are critical.

There are some study limitations that necessitate explanation. First, self-reported PA data is subject to recall and social desirability biases. While social desirability bias has been associated with an over-estimation of PA in more developed countries [54,55], less is known about the potential for social desirability bias in developing countries where the benefit of PA and stigma of overweight is less widespread. Furthermore, the PA data collection methods in children varied slightly by age-group, with parents or caregivers assisting with survey completion for children <10 years. While the varying data collection methods were used for improved accuracy of child PA data [56-58], comparison of CHNS child PA data based on parent-assisted self-report (children <10 years) and self-report (children ≥10 years) has not yet been conducted to the best of our knowledge.. Another limitation is the inability of the MET-hrs/wk measurement to consider individual differences in energy expenditure associated with the same activities. However, the use of the MET remains the most appropriate means for estimating energy costs associated with self-reported physical activities [59] and our application of adult and youth compendiums ensures the most accurate comparison of mother and child PA patterns. Our inclusion criteria and cohort determination resulted in a smaller sample population, which may have limited our ability to detect cohort or additional socioeconomic effects on PA/inactivity patterns. Lastly, our 2–4 year time frame was a relatively short period of time to observe PA changes.

Our study was unique in longitudinally comparing PA patterns of mothers versus children over time. A major strength of the CHNS is use of the same data collection tools for the assessment of PA in mothers and children, and our use of adult and child compendiums provided examination of PA measures in mothers versus children with improved accuracy. While previous CHNS investigators have separately studied PA patterns and their relationship with urbanization or socioeconomic factors in adults and children [3,4,34,60], our study was the first to systematically compare parent–child PA patterns and to investigate how PA evolves over periods of rapid socioeconomic growth in China.

Continued emphasis on PA promotion among Chinese youth is needed to further increase and maintain child PA as they age, while concerted efforts to improve PA among Chinese adults are also necessary. Additional initiatives to prevent further increases and reduce existing levels of sedentary behavior in mothers and children are crucial. Focusing PA promotion at the family-level could increase the public health impact and effectiveness of these interventions [61-63]. The success of these efforts will continue to be challenged by rapid urbanization, technological advances, and socioeconomic development, so PA policy and interventions must be cognizant of how these factors influence PA and inactivity patterns. Future research integrating PA and dietary intake patterns is needed to better understand how these patterns relate to overweight and obesity in adults versus children.

## Competing interests

The authors declare that they have no competing interests.

## Authors’ contributions

TDW and BMP conceived the study, TDW conducted data analyses, TDW, PGL and BMP wrote the manuscript, and LSA and BZ provided significant advice or consultation. All authors have read and approved the final manuscript.
